# N-Acetylcysteine Regenerates In Vivo Mercaptoalbumin

**DOI:** 10.3390/antiox11091758

**Published:** 2022-09-06

**Authors:** Alessandra Anna Altomare, Maura Brioschi, Sonia Eligini, Alice Bonomi, Beatrice Zoanni, Ada Iezzi, Costantino Jemos, Benedetta Porro, Yuri D’Alessandra, Anna Guarino, Emanuela Omodeo Salè, Giancarlo Aldini, Piergiuseppe Agostoni, Cristina Banfi

**Affiliations:** 1 Department of Pharmaceutical Sciences, University of Milan, 20133 Milano, Italy; 2 Unit of Functional Proteomics, Metabolomics and Network Analysis, Centro Cardiologico Monzino IRCCS, 20138 Milano, Italy; 3 Centro Cardiologico Monzino IRCCS, 20138 Milano, Italy; 4 Division of Pharmacy, IEO European Institute of Oncology IRCCS, 20141 Milano, Italy; 5 Cardiovascular Tissue Bank of Milan, Centro Cardiologico Monzino IRCCS, 20138 Milano, Italy; 6 Cardiovascular Section, Department of Clinical Sciences and Community Health, University of Milan, 20122 Milano, Italy

**Keywords:** s-thiolation, mercaptoalbumin, albumin, oxidative stress, mass spectrometry

## Abstract

Human serum albumin (HSA) represents the most abundant plasma protein, with relevant antioxidant activity due to the presence of the sulfhydryl group on cysteine at position 34 (Cys34), the latter being one of the major target sites for redox-dependent modifications leading to the formation of mixed disulfide linkages with low molecular weight thiols. Thiolated forms of HSA (Thio-HSA) may be useful as markers of an unbalanced redox state and as a potential therapeutic target. Indeed, we have previously reported that albumin Cys34 can be regenerated in vitro by *N*-Acetylcysteine (NAC) through a thiol-disulfide breaking mechanism, with a full recovery of the HSA antioxidant and antiplatelet activities. With this case study, we aimed to assess the ability of NAC to regenerate native mercaptoalbumin (HSA-SH) and the plasma antioxidant capacity in subjects with redox unbalance, after oral and intravenous administration. A placebo-controlled crossover study, single-blinded, was performed on six hypertensive subjects, randomized into two groups, on a one-to-one basis with NAC (600 mg/die) or a placebo, orally and intravenously administered. Albumin isoforms, HSA-SH, Thio-HSA, and glutathione levels were evaluated by means of mass spectrometry. The plasma antioxidant activity was assessed by a fluorimetric assay. NAC, orally administered, significantly decreased the Thio-HSA levels in comparison with the pre-treatment conditions (T0), reaching the maximal effect after 60 min (−24.7 ± 8%). The Thio-HSA reduction was accompanied by a concomitant increase in the native HSA-SH levels (+6.4 ± 2%). After intravenous administration of NAC, a significant decrease of the Thio-HSA with respect to the pre-treatment conditions (T0) was observed, with a maximal effect after 30 min (−68.9 ± 10.6%) and remaining significant even after 6 h. Conversely, no effect on the albumin isoforms was detected with either the orally or the intravenously administered placebo treatments. Furthermore, the total antioxidant activity of the plasma significantly increased after NAC infusion with respect to the placebo (*p* = 0.0089). Interestingly, we did not observe any difference in terms of total glutathione corrected for hemoglobin, ruling out any effect of NAC on the intracellular glutathione and supporting its role as a disulfide-breaking agent. This case study confirms the in vitro experiments and demonstrates for the first time that NAC is able to regenerate mercaptoalbumin in vivo, allowing us to hypothesize that the recovery of Cys34 content can modulate in vivo oxidative stress and, hopefully, have an effect in oxidative-based diseases.

## 1. Introduction

Human serum albumin (HSA) represents the most abundant protein in the plasma, with relevant antioxidant activity due to the presence of the sulfhydryl group on cysteine at position 34 (Cys34). Indeed, HSA contains 35 cysteine residues, 34 of which form disulfide bridges, whilst only Cys34 exists as a free sulfhydryl group [1]. The latter constitutes the largest pool of free thiols in the circulation and works as a radical scavenger thanks to its peculiar acidity and solvent accessibility [2]. Cys34 is also one of the major target sites for redox-dependent modifications in HSA through the formation of mixed disulfide linkages with low-molecular-weight thiols [3]. Thus, due to the high abundance and long half-life of HSA in blood, thiolated forms of HSA (Thio-HSA) may be useful for the characterization of the (patho)physiological conditions linked to an unbalanced redox state. Furthermore, S-thiolation at the Cys34 residue may have profound implications on the structural and functional properties of HSA [4]. In this regard, we have previously shown that Thio-HSA increases in the plasma of patients with heart failure; it is inversely associated with the parameters of disease severity, and it does not protect cardiomyocytes from the oxidant agent hydrogen peroxide [5]. Furthermore, Thio-HSA has an impaired platelet anti-aggregant activity, thus likely contributing to the progression of cardiovascular diseases [6]. In addition, we have previously reported that albumin Cys34 can be regenerated in vitro by the dietary supplement *N*-Acetylcysteine (NAC) through a thiol-disulfide breaking mechanism [7], with a full recovery of the HSA antioxidant and antiplatelet activities [6,7]. NAC is commonly used, in the clinical practice, as a mucolytic [8] and a detoxifying agent against acetaminophen poisoning [9]. However, its clinical uses are expanding in diseases characterized by oxidative stress [10], spanning, for example, from renal protection in contrast-induced nephropathy [11] and atrial fibrillation [12] to the treatment of psychiatric and neurological disorders [13]. Despite the efficacy of NAC being mainly attributed to its ability to induce the intracellular biosynthesis of glutathione (GSH), a well-known direct antioxidant and substrate for several antioxidant enzymes [14], all the mechanisms underlying its activity are not yet fully understood in that they may not depend exclusively on GSH replenishment [15].

With this pilot study, we aimed to assess the ability of NAC, orally and intravenously administered, to regenerate native mercaptoalbumin (HSA-SH) and the plasma antioxidant capacity in subjects with redox unbalance, in order to provide data and rationale for the design of further trials with a widespread indication of NAC administration to reduce Thio-HSA.

## 2. Methods

### 2.1. Study Design

**Figure 1:**
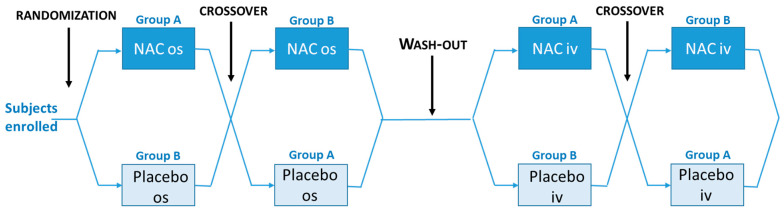
Workflow of the study.

A placebo-controlled crossover study, single-blinded, was performed on 6 hypertensive subjects, randomized in two groups, on a one-to-one basis with NAC (600 mg/die) or placebo (Figure 1), orally administered. Indeed, hypertension is a condition associated with oxidative stress [16]. Blood was collected at different time points (basal, 30 min, 1 h, 2 h, 4 h, 6 h, and 24 h after NAC or placebo administration) to evaluate the time-dependent effect of NAC on the reduction of Thio-HSA and on the redox balance. After one month of wash-out, the two groups were crossed over. The same scheme described above was applied to the same 6 patients treated with NAC or placebo, by intravenous infusion (i.v.) (slowly infused for 10 min, diluted in 20 mL of saline solution), after the wash-out period.

The study inclusion criteria were age >18 years old, body weight greater than 45 kg, and hypertension (according to the current guidelines for high blood pressure of the American Heart Association and the American College of Cardiology; systolic blood pressure >130 and diastolic blood pressure >80 mm Hg). Th exclusion criteria were positive to pregnancy test, severe obstructive and/or restrictive lung disease, anemia (hemoglobin, 11 g/dL), history and/or documentation of pulmonary embolism, any acute or serious comorbid condition (e.g. major infection or hematologic, cardiac, renal, metabolic, cerebrovascular, gastrointestinal, or endocrine dysfunction), history of clinically significant malignant diseases, hypersensitivity to the active substance or to any of the excipients, administration of an experimental drug within 30 days or 5 half-lives of the investigational drug. The study was approved by the Ethical Committee of Centro Cardiologico Monzino IRCCS (R 1250/20 CCM 1140, 7 October 2020; EudraCT 2019-004188-35) and was conducted according to the Declaration of Helsinki. Written informed consent was obtained from all the patients.

### 2.2. Plasma Preparation

Blood was drawn into Vacutainer tubes containing sodium citrate 0.129 mol/L as an anticoagulant and immediately prepared by centrifugation at 1500× *g* for 15 min at 4 °C.

### 2.3. Albumin Analysis by Mass Spectrometry

The plasma samples were diluted 40-fold in H_2_O/CH_3_CN/HCOOH (70:30:0.2), which is the initial condition of the chromatographic gradient. The diluted samples were then filtered with 0.45 µm filters to remove any particulate, transferred into vials, and injected in triplicate into the liquid chromatography-mass spectrometry (LC-MS) system.

Thio-HSA and HSA-SH were measured by MS intact protein analysis. The analyses were performed on an ExionLC-100 coupled to an API4000 equipped with a Turbo-V ESI source (AB-Sciex), using the chromatographic conditions reported by Altomare et al. [7]: plasma proteins were separated on a reversed-phase Phenomenex LC column Jupiter-C4 (150 × 2 mm, i.d. 5 µm, 300 Å, Milan, Italy), protected by a Phenomenex security-guard column. Briefly, 10 µL of diluted plasma were injected, and the analytes were eluted with a 20 min multi-step gradient of phase A, H_2_O: HCOOH (100:0.1% *v:v*) and phase B, CH_3_CN: HCOOH (100:0.1% *v:v*): 0–2 min, isocratic of 30% phase B; 2–13 min, linear gradient from 30% phase B to 50% phase B; 13–14 min, linear gradient from 50% phase B to 95% phase B; 14–17 min, of isocratic 95% phase B; and then 3 final minutes of isocratic 30% phase B to re-equilibrate the column. The mass spectrometer was set to acquire a full MS spectrum (full scan mode) on the Q3 quadrupole by selecting the mass range *m*/*z* 1000–1500 (Q = *m*/*z* 1) and setting the following experimental ionization source parameters (ESI): positive ion mode; spray voltage: 4000 volts; source temperature: 450 °C; gas 1: 40; gas 2: 50; curtain gas: 25; declustering potential: 100; and EP: 10. The instrument control was carried out with Analyst (version 1.6.3; AB-Sciex). Spectra deconvolution and calculation of the relative abundance of Thio-HSA and native HSA-SH were performed using the MagTran software (Mag-Tran 1.03b2) set as follows: max peak width = 45 Da; min peak width = 5 Da; max iteration = 3192; Rho = 0.99; max no. of Gaussian = 3/5.

### 2.4. Plasma Antioxidant Capacity Analysis (TRAP Assay)

The plasma antioxidant activity was assessed by a fluorimetric assay based on 2′,7′-dichlorodihydrofluorescein diacetate (DCFH_2_-DA) and 2,2′-Azobis(2-amidinopropane) dihydrochloride (AAPH) as substrate and radical initiator, respectively [17].

### 2.5. Measurement of Glutathione

Reduced (GSH) and oxidized glutathione (GSSG) levels were measured in the blood by means of an LC-MS/MS method previously developed by us [18]. The analysis was performed by means of an Accela chromatographic system coupled with a triple quadrupole mass spectrometer TSQ Quantum Access (Thermo Fisher Scientific, Rodano, Milan, Italy) with an electrospray ionization source in positive ion mode. The transitions monitored were *m*/*z* 308.1 → *m*/*z* 76.2 + 84.2 + 161.9 for GSH and *m*/*z* 613.2 → *m*/*z* 230.5 + 234.6 + 354.8 for GSSG. The data were obtained by comparison with calibration curves using GSH and GSSG standard solutions (Sigma-Aldrich, Merck Life Science S.r.l., Milan, Italy). The intra- and inter-day CVs % were <5%, and the limits of detection were 0.031 µmol/L and 0.008 µmol/L for GSH and GSSG, respectively. The levels of GSH and GSSG were corrected for hemoglobin (Hb) and expressed as µmol/g Hb.

### 2.6. Statistical Analysis

Continuous data were expressed as mean and standard deviation (SD). Categorical data were expressed as frequency and percentage. The data are graphically represented as mean and standard error (SEM). ANOVA for repeated measures with Dunnett’s post-hoc test was used to analyze differences with respect to the values obtained before treatment (T0). The Student’s t test was used to compare two conditions. Two-way ANOVA was used to determine the effect of the treatment. Linear mixed-effects models for repeated measures were used to investigate the cross-sectional and longitudinal trends of each outcome on the treatment, after controlling for the effect of time and their interaction, treatment order, and administration type. Then, an analysis was repeated, stratifying by administration type. All the results were presented as two-tailed values, with a *p* value  <  0.05 considered as statistically significant. Statistical analyses were performed by SAS Software (version 9.4; SAS Institute Inc., Cary, NC, USA) and GraphPad Prism (version 5).

## 3. Results

In this crossover study, we enrolled six patients with hypertension (systolic blood pressure 133 ± 10.8 mm Hg, diastolic blood pressure 87 ± 8.2 mm Hg), randomized to receive NAC or placebo, orally and intravenously. The patients were 50% females; the mean age was 53.83 ± 7.7 years; three patients were treated with anti-hypertensive drugs and two patients with statins.

The albumin isoforms, including native HSA (HSA-SH) and thiolated HSA (Thio-HSA), were evaluated at different time points from treatment by intact protein analysis (LC-MS).

**Figure 2:**
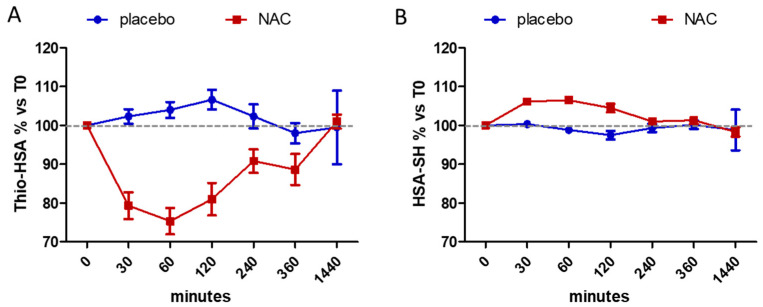
Effects of oral administration of NAC or placebo on albumin isoforms, Thio-HSA (**A**) and HSA-SH (**B**). Data are expressed as mean ± SEM of the percentage with respect to T0 values. Two-way ANOVA was used to assess the effect of treatment (*p* = 0.0006 and *p* = 0.027 for Thio-HSA and HSA-SH, respectively).

As shown in Figure 2 and Table 1, the oral administration of NAC results in a statistically significant decrease of the Thio-HSA with respect to the pre-treatment conditions (T0), with a maximal effect after 60 min (−24.7 ± 8%) and a concomitant increase of the native HSA-SH (+6.4 ± 2%). Conversely, no effect on the albumin isoforms was detected with the placebo treatment. The representative deconvoluted MS spectra of HSA isoforms (66,472 Da and 66,592 Da, for HSA-SH and Thio-HSA, respectively) are shown in Supplementary Figure S1.

**Figure 3:**
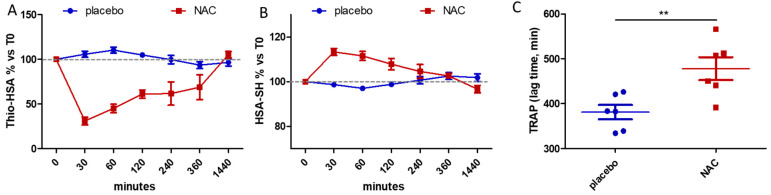
Effects of intravenous administration of NAC or placebo. Mass spectrometry evaluation of albumin isoforms, Thio-HSA (**A**) and HSA-SH (**B**), at different time points from NAC or placebo administration. Data are expressed as mean ± SEM of the percentage with respect to T0 values. Two-way ANOVA was used to assess the effect of treatment (*p* < 0.0001 for both Thio-HSA and HSA-SH). (**C**) Total plasma antioxidant activity measured by TRAP assay after 30 min from NAC or placebo administration. Data are presented as circle plot, with each circle representing an individual patient and bars showing the mean value ± SEM. ** *p* < 0.01 calculated by Student’s *t* test.

As shown in Table 2 and Figure 3, after intravenous administration of NAC we evidenced a significant decrease in the Thio-HSA with respect to the pre-treatment conditions (T0), with an earlier maximal effect after 30 min (−68.9 ± 10.6% vs. T0), remaining significant even after 6 h, and a concomitant increase in the native form of HSA-SH (+13.4 ± 3.6% vs. T0 after 30 min), while no effect on the albumin isoform distribution was detected with the placebo treatment. The representative deconvoluted MS spectra of the HSA isoforms are shown in Supplementary Figure S2.

Of note, in correspondence with the maximum reduction of Thio-HSA, after 30 min from infusion, the total antioxidant activity of plasma significantly increased after the NAC treatment with respect to the placebo (*p* = 0.0089) (Figure 3C). Interestingly, considering that NAC is the precursor of intracellular reduced glutathione (GSH), we measured the levels of GSH in the blood before treatment and after NAC administration, both orally and intravenously, but we did not observe any difference in terms of reduced GSH corrected for hemoglobin (4.49 ± 2.10 µmol/g Hb and 3.69 ± 1.56 µmol/g Hb with oral administration at T0 and after 4 h, respectively; 4.04 ± 1.09 µmol/g Hb and 3.43 ± 1.11 µmol/g Hb with intravenous administration at T0 and after 4 h, respectively) or oxidized GSSG (0.95 ± 0.17 µmol/g Hb and 1.09 ± 0.29 µmol/g Hb with oral administration at T0 and after 4 h, respectively; 1.02 ± 0.27 µmol/g Hb and 1.19 ± 0.45 µmol/g Hb with intravenous administration at T0 and after 4 h, respectively).

Performing a multivariable analysis, including the route of administration and the administration sequence, we observed a significant difference in the HSA-SH between the treatments (*p* = 0.03), a significant effect of the route of administration (*p* = 0.001) and administration sequence (*p* = 0.0473). In addition, the interaction between time and treatment on the HSA-SH was statistically significant (*p* = 0.0105).

Considering Thio-HSA, we detected a significant effect of the route of administration (*p* = 0.0327) and treatment (*p* = 0.0068), and the interaction effect of time and treatment was also significant (*p* = 0.0009).

Stratifying by the route of administration, we detected a significant effect of treatment (*p* = 0.01 for HSA-SH and *p*= 0.0003 for Thio-HSA) and a significant interaction between time and treatment (*p* = 0.003 for HSA-SH and *p* < 0.0001 for Thio-HSA) only in the intravenous administration.

Finally, no adverse events are reported in this study.

## 4. Discussion

This case study, performed on six subjects with redox unbalance, confirms the previous in vitro experiments [7] and demonstrates for the first time that NAC is able to regenerate mercaptoalbumin in vivo. This effect is more pronounced after intravenous administration, and it is concomitant with the recovery of the antioxidant capacity of albumin. The observation that the blood glutathione levels were not affected by NAC during the experimental time frame is consistent with its main activity as a disulfide-breaking agent [15]. Indeed, in a recent review [15], we critically reviewed the main molecular mechanisms explaining the well-established antioxidant activity of NAC. As far as its direct antioxidant activity is concerned, it emerges that the reaction rate of the drug and other thiols (i.e. cysteine and glutathione) is negligible in comparison with that of endogenous antioxidant enzymes, such as peroxiredoxins [15]. It means that, even at a high plasma concentration (100 μmol/L), which is obtained after four doses of 2 g of NAC [19], the reaction between it and the oxidant hydrogen peroxide is 5.6 × 10^6^ slower than, for example, the GSH peroxidase 3. Similar considerations can be made with other oxidants, leading to the conclusion that a direct antioxidant activity of NAC can be ruled out for hydrogen peroxide, hydroxyl radical, superoxide anion, and peroxynitrous acid, whilst it can be plausible for others, such as nitrogen dioxide and hypohalous acid [15].

Furthermore, NAC can exploit its antioxidant activity indirectly by boosting the formation of intracellular GSH as a precursor of cysteine, the rate-limiting factor in cellular GSH synthesis. This process occurs after the deacetylation of NAC by tissue aminoacylases [20], which are particularly active in the kidneys. Thus, after the oral administration of 600 or 1200 mg/kg/day NAC for 30 days, the levels of GSH significantly increased in the kidneys and in other tissues [21]. In the blood, it has been shown that GSH levels increased at 8 days after NAC administration [22]. Thus, given the rapid effect of NAC on albumin proteoforms, we exclude an effect of NAC mediated by GSH.

Cys34 on HSA is the main plasma antioxidant due not only to its high content (0.6 mmol/L) but also to the high reactivity of the SH group, explained by its peculiar acidity given by a particular microenvironment able to stabilize the thiolate anion. The acid property of Cys34 [(pKa = 8.1, significantly higher than that of Cys (pKa = 8.6) and GSH (pKa = 9.2)] causes the equilibrium between the thiolate anion and the undissociated thiol form to shift toward the former, which is the active form able to react with oxidants and electrophilic species [23].

The thiolate anion of Cys34 detoxifies radical and non-radical oxidant species, including ROS and RNS, such as hydrogen peroxide (H_2_O_2_), peroxynitrite (ONOO^–^), nitric oxide, (NO^•^), superoxide (O_2_^•–^), and hypochlorous acid (HOCl) [24]. When Cys34 acts as an antioxidant, the corresponding sulfenic acid derivative is formed, which then reacts with free Cys (the main extracellular low-molecular thiol), forming the corresponding mixed disulfide (cysteinylation reaction) and thus preserving the irreversible oxidation of Cys34 to sulfinic and sulfonic acid derivatives [25]. Indeed, we confirmed that Thio-HSA is due to the binding of cysteine that represents almost 90% of the low-molecular-weight thiols bound to HSA [5]. Hence, the cysteinylation of Cys34, while impairing the antioxidant activity of Cys34, also prevents the formation of irreversible modifications. The cysteinylated form is then presumably reversed to Cys34 through an endogenous pathway breaking the mixed disulfide, whose molecular mechanism and kinetic are still unresolved.

Besides acting as a direct antioxidant, Cys34 also reacts with electrophilic reactive carbonyl species (RCS) compounds, such as acrolein and 4-hydroxy-nonenal, arising from the oxidative degradation of biomolecules [23,26]. RCS can act as damaging compounds, and their overproduction in plasma has been related to different diseases, including atherosclerosis [27,28]. Hence, albumin Cys34 is the main detoxifying agent of plasma oxidants and RCS, and, taking into account their pathogenetic effects, Cys34 should act as a key element in reducing the onset and progression of some oxidative-based diseases [23,29].

The in vivo antioxidant role of Cys34 is confirmed, although indirectly, by several studies reporting a relationship between the Cys34 reduction/cysteinylation increase and the severity of chronic diseases for which oxidative stress is a significant etiological manifestation, including chronic liver disease, kidney diseases, diabetes mellitus [30], muscle dystropathology [31], and heart failure [5]. Moreover, the adducts of Cys34 with electrophilic reaction products have been reported to increase in ischemia-reperfusion injury during hepatectomy [32] and in colorectal cancer patients [33]. Hence, Cys34′s oxidative status and the formation of Michael adducts are currently reported as a promising biomarker of oxidative stress [34,35], and Cys34 can be considered as a bioactive protectant involved in the redox balance of plasma.

However, many aspects still need to be clarified to better understand the role of Cys34 as an antioxidant. Currently, the endogenous mechanisms and players devoted to the breaking of the mixed disulfides leading to the endogenous regeneration of Cys34 are unknown. Moreover, drugs or pharmacological tools able to regenerate Cys34 from the cysteinylated derivatives are not yet reported, and this greatly limits a better understanding of the molecular mechanisms of Cys34, its role as an antioxidant, and whether Cys34 regeneration could be a promising pharmacological target.

We have recently found in in vitro studies that NAC is a suitable compound able to break the HSA-Cys bond, forming the dimer NAC-Cys and regenerating Cys34, resulting in an improvement in the plasma antioxidant activity [7,15]. In the present work, we found that NAC, at a dose of 600 mg/Kg, partially restores Cys34 by breaking the cysteinylated albumin, and this represents, to our knowledge, the first report on the recovery of Cys34 by a drug in in vivo conditions. In particular, after the i.v. bolus of 600 mg of NAC, corresponding to 3.676 mmoles (MW 163.2), we observed, within 30 min, a reduction of the cysteinylated albumin (Thio-HSA) of almost 13%. By considering a physiological concentration of HSA of 0.6 mmol/L, the amount of cysteinylated HSA undergoing the disulfide breaking accounts for 78 µmol/L. The concentration of NAC given at a dose of 600 mg by i.v. bolus is reported to be 36.1 mg/L (220 µmol/L) [36]; therefore, we can calculate that almost 3 moles of NAC are required to reduce 1 mole of cysteinylated albumin when given by i.v. bolus. An almost overlapping stoichiometry was observed in previous in vitro experiments where a slope of 0.2 was found by plotting the nmoles/mL of NAC vs. the nmoles of reduced cysteinylated albumin, indicating a 5:1 stoichiometric ratio for Cys34-Cys breaking by NAC [7].

The disulfide breaking of Thio-HSA was accompanied by a 9% increase of HSA-SH after 30 min, which time-dependently reduced, together with a concomitant increase of the cysteinylated form, reaching the pre-treatment content within 24 h. The partial restoration of HSA-SH peaking after 30 min of infusion was also related to a significant increase in the radical scavenging activity of the plasma which, as previously reported [7], is not related to circulating NAC, but to the partial restoration of Cys34.

The fact that the plasma content of the HSA-SH and of the corresponding Thio-HSA forms returns to the basal level after 24 h suggests a steady oxidative stress that is only temporarily perturbed by the bolus of NAC and that is neutralized within 24 h. A stimulating challenge is to understand whether a chronic administration of NAC can change the basal content of HSA-SH, thus improving the reducing condition.

To investigate the effect of a chronic NAC treatment on HSA-Cys breaking, which is clearly not affordable by an i.v. infusion, we then tested the efficacy of an equimolar dose of NAC given by oral administration. NAC has a limited bioavailability, which is reported to be almost 10% in both healthy and intensive care unit patients [36,37]. The plasma concentration of NAC after a bolus of 600 mg is 36.1 µg/mL [36], while after an oral dose of an equal amount, it reaches a Cmax of 2.5 µg/mL with a Tmax of 1–2 h [38,39]. Based on the limited oral bioavailability of NAC, we expected to detect an effect of orally administered NAC much lower than that observed after the i.v. infusion (almost ten times lower, according to the plasma levels of NAC). Moreover, by considering the extent of the effect, and the biological variability, the lack of a significant effect was also taken into account. Surprisingly, we observed a significant reduction of the Thio-HSA of 4.4%, and an increase of the HSA-SH of almost 4%, indicating that the effects of the oral treatment on Thio-HSA breaking and HSA-SH regeneration are reduced only 2–3 fold with respect to the i.v. infusion (13% and 9%, respectively). Hence, the effects observed after an oral administration were found to be clearly reduced with respect to the i.v. but were much higher than expected, on the basis of the bioavailability and concentrations. Several explanations can be taken into account, among which is the slower kinetic of NAC absorption when orally given (the Tmax ranges between 1 and 2 h). The results obtained with the oral administration will permit us to investigate whether a chronic oral absorption of NAC can consistently increase the basal content of HSA-SH, thus affecting the steady oxidative status of plasma. If such an effect is found in hypertensive subjects, the chronic treatment of patients affected by oxidative-based diseases will permit the assessment of the effect of shifting the redox balance on the progression of the disease. In addition to the effect of Cys 34 on redox balance, its regeneration by a pharmacological treatment would also have other benefits, as albumin cysteinylation affects the HSA binding towards xenobiotics and endogenous ligands. In particular, the affinity of HSA for the endogenous ligands bilirubin and tryptophan, as well as for the exogenous pharmaceuticals warfarin and diazepam, decreases in proportion to the level of oxidized albumin (cysteinylation of Cys34) [30]. The affinity for lipids also differs: pro-atherosclerotic lysophosphatidylcholine and lysophosphatidic acid have a higher affinity for the oxidized isoform, while the anti-atherosclerotic derivatives of eicosapentaenoic and docosahexaenoic acids have a higher affinity for the reduced isoform of albumin [40].

In conclusion, we demonstrated that NAC, when given either by i.v. or oral treatment, is able to regenerate Cys34 by breaking the disulfide bond between Cys34 and Cys, resulting in an increased antioxidant plasma activity. Finally, no adverse events are reported in this study. Indeed, according to meta-analysis, NAC (used for various indications) does not significantly increase the risk of side effects when compared to a placebo. Overall, the clinical data reveal that NAC has a benign and tolerable side effect profile [41,42].

Finally, to the best of our knowledge this is the first study in humans reporting the efficacy of a pharmacological treatment in reducing Thio-HSA in favor of HSA-SH. These results will prompt us to test whether chronic treatment with NAC is able to increase the basal level of Cys34. Such a treatment will permit us to establish whether the recovery of Cys34 content can modulate in vivo oxidative stress and have an effect on oxidative-based diseases.

## Figures and Tables

**Table 1:** Effects of oral administration of NAC or placebo on albumin isoforms.

	Placebo		NAC	
Minutes	Thio-HSA	HSA-SH	Thio-HSA	HSA-SH
0	21.50 ± 3.30	60.10 ± 5.22	20.70 ± 4.31	60.97 ± 3.98
30	21.97 ± 3.32	60.15 ± 4.29	16.28 ± 3.23 ^§^	64.63 ± 3.43 ^§^
60	22.37 ± 3.43	59.43 ± 5.27	15.75 ± 4.60 ^§^	64.85 ± 3.33 ^§^
120	22.98 ± 3.95	58.62 ± 5.30	17.08 ± 5.56 ^#^	63.78 ± 4.47 ^#^
240	21.92 ± 3.03	59.58 ± 4.94	18.98 ± 5.18	61.62 ± 4.68
360	21.12 ± 4.12	60.30 ± 6.23	18.43 ± 4.85	61.72 ± 4.14
1440	21.17 ± 5.46	59.48 ± 9.135	20.98 ± 4.93	60.02 ± 4.45

Data are expressed as mean ± SD; § *p* < 0.001 vs. T0; # *p* < 0.01 vs. T0, ANOVA for repeated measures with Dunnett’s post-hoc test.

**Table 2:** Effects of intravenous administration of NAC or placebo on albumin isoforms.

	Placebo		NAC	
Minutes	Thio-HSA	HSA-SH	Thio-HSA	HSA-SH
0	20.05 ± 1.96	65.40 ± 1.92	18.73 ± 1.72	66.65 ± 2.59
30	21.15 ± 1.92	64.58 ± 2.04	5.73 ± 1.69 ^§^	75.52 ± 3.46 ^§^
60	21.98 ± 1.65	63.42 ± 2.00	8.37 ± 1.73 ^§^	74.35 ± 3.78 ^#^
120	20.98 ± 1.96	64.83 ± 3.14	11.37 ± 1.86 ^§^	71.67 ± 3.44
240	19.87 ± 1.81	65.82 ± 3.31	13.80 ± 1.92 ^§^	69.32 ± 4.08
360	18.80 ± 2.81	67.13 ± 4.08	15.46 ± 1.52 *	67.84 ± 2.65
1440	19.37 ± 2.74	66.60 ± 4.40	19.73 ± 2.36	64.35 ± 3.68

Data are expressed as mean ± SD; § *p* < 0.001 vs. T0; # *p* < 0.01 vs. T0; * *p* < 0.05 vs. T0, ANOVA for repeated measures with Dunnett’s post-hoc test.

## Data Availability

Data collected in the study will be made available using the data repository Zenodo (accessed on 5 September 2022, https://zenodo.org/), with restricted access upon request to direzione.scientifica@ccfm.it.

## Supplementary Materials

The following supporting information can be downloaded at: https://www.mdpi.com/article/10.3390/antiox11091758/s1, Figure S1: effects of oral administration of NAC (left panels) and placebo (right panels) on albumin isoforms, Figure S2: effects of intravenous administration of NAC (left panels) and placebo (right panels) on albumin isoforms.
