# Supplementary material for: N-Acetylcysteine Regenerates In Vivo Mercaptoalbumin
Source: Antioxidants (Basel). 2022 Sep 6;11(9):1758. doi: 10.3390/antiox11091758 (PMC9495570; doi:10.3390/antiox11091758)
Supplement: Supplementary file 1 [file antioxidants-11-01758-s001.zip › antioxidants-1893292-supplementary.pdf]

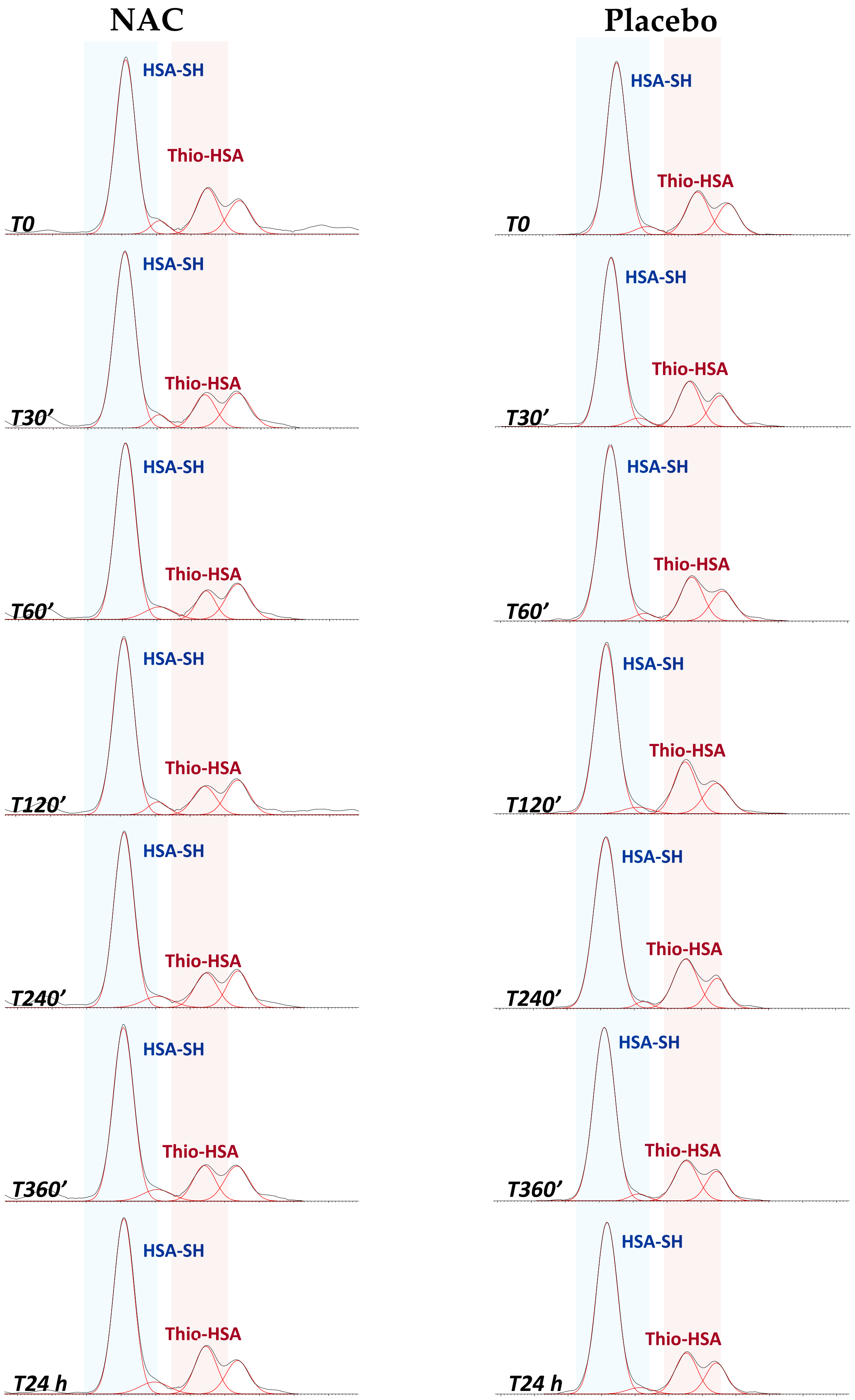

Figure S1. Effects of oral administration of NAC (left panels) and placebo (right panels) on albumin isoforms. Deconvoluted spectrum of HSA proteoforms showing the mercaptoalbumin (HSA-SH) at 66472 Da, and the thiolated form, Thio-HSA, at 66592 Da (+120 Da with respect to HSA-SH).

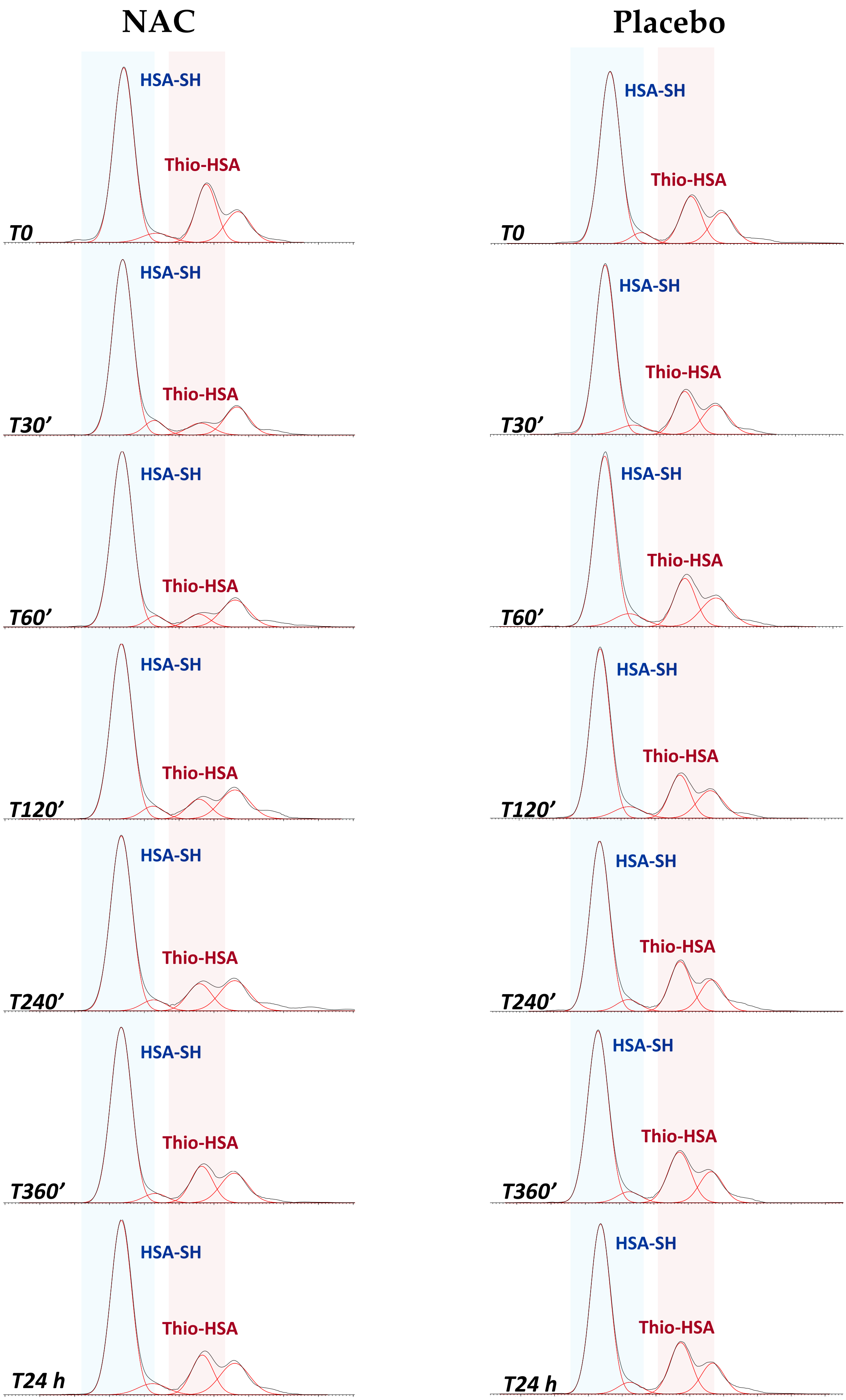

Figure S2. Effects of intravenous administration of NAC (left panels) and placebo (right panels) on albumin isoforms. Deconvoluted spectrum of HSA proteoforms showing the mercaptoalbumin (HSA-SH) at 66472 Da, and the thiolated form, Thio-HSA, at 66592 Da (+120 Da with respect to HSA-SH).
